# Genetic variation in *PLAG1* is associated with early fertility in Australian Brahman cattle

**DOI:** 10.1093/jas/skac084

**Published:** 2022-03-16

**Authors:** Bailey N Engle, Ben J Hayes

**Affiliations:** Queensland Alliance for Agriculture and Food Innovation, University of Queensland, St Lucia, QLD 4072, Australia

**Keywords:** Brahman, heifer fertility, *PLAG1*, stayability

## Abstract

Variation in the genome region coding for *PLAG1* has well-documented associations with skeletal growth and age at puberty in cattle. However, the influence of *PLAG1* on other economically important traits such as cow stayability has not yet been explored. Here we investigate the effect of *PLAG1* variation on early and later in life female fertility, as well as size and growth, in a well-phenotyped Australian Brahman herd. Yearly pregnancy and productivity records were collected from 2,839 genotyped Brahman cows and used to generate fertility, growth, and weight phenotypes. A variant on chromosome 14 in *PLAG1* (NC_037341.1:g.23338890G>T, rs109815800) was previously determined to be a putative causative mutation associated with variation in cattle stature. The imputed *PLAG1* genotype at this variant was isolated for each animal and the effect of *PLAG1* genotype on each trait was estimated using linear modeling. Regardless of how heifer fertility was measured, there was a significant (*P* < 0.05) and desirable relationship between the additive effects of *PLAG1* genotype and successful heifer fertility. Heifers with two copies of the alternate allele (TT) conceived earlier and had higher pregnancy and calving rates. However, the effects of *PLAG1* genotype on fertility began to diminish as cows aged and did not significantly influence stayability at later ages. While there was no effect of genotype on growth, *PLAG1* had a negative effect on mature cow weight (*P* < 0.01), where females with two copies of the alternate allele (TT) were significantly smaller than those with either one or none. Selection emphasis on improved Brahman heifer fertility will likely increase the frequency of the T allele of rs109815800, which may also increase herd profitability and long-term sustainability through improved reproductive efficiency and reduced mature cow size.

## Teaser

An allele of *PLAG1* genotype had a significant and positive relationship with Brahman heifer fertility, although the effect on fertility diminished as cows aged. While there was no effect of *PLAG1* genotype on growth, there was a significant and negative relationship of the allele improving heifer fertility with mature cow weight.

## Introduction

Puberty is the first critical milestone in a beef cow’s reproductive life. Brahman cattle, and other *Bos indicus* breeds, tend to be older at the onset of puberty than their taurine counterparts. This is speculated to be an adaptive response allowing them to better withstand challenging environments (Rodrigues et al., 2002; Low et al., 2020). The average range that Brahmans reach puberty is reported to span anywhere between 582 d (Plasse et al., 1968) and 751 d (Johnston et al., 2009). In modern beef cattle production, this large range often results in management difficulties and the potential for economic losses. Despite its importance, the underlying genetic causes driving differences in age at puberty are poorly understood.

Age at puberty in Brahman and other *Bos indicus-*influenced beef females has previously been shown to be highly polygenic. Genome-wide association studies have identified many genes and gene networks that partially explain this variation (Fortes et al., 2010; Fortes et al., 2012; Hawken et al., 2012; Canovas et al., 2014). Variation in the pleomorphic adenoma gene 1 (*PLAG1*) has been consistently shown to explain a large proportion of variance associated with age at puberty in female Brahman cattle (Hawken et al., 2012; Fortes et al., 2013).

*PLAG1* encodes a developmentally regulated zinc finger protein. The *PLAG1* gene is a versatile transcription factor known to regulate many genes and pathways, among them, it has been shown to initiate transcription of *IGF2* (Voz et al., 2000; Van Dyck et al., 2007) and *IGF1R* (Van Dyck et al., 2007). Within cattle, *PLAG1* appears to be ubiquitously expressed (Nguyen et al., 2017; Li et al., 2020), generally at a very low level within adult animals, but with a significant increase in expression in fetal tissues from a variety of species (Hensen et al., 2004; Li et al., 2020).

*PLAG1* variants have also been associated with height or skeletal size in a number of species, including humans, cattle, and mice (Hensen et al., 2004; Karim et al., 2011; Pryce et al., 2011; Fortes et al., 2013; Wood et al., 2014; Utsunomiya et al., 2017; Bouwman et al., 2018). For cattle, Karim et al. (2011) was the first to identify a quantitative trait locus on chromosome 14 with a major effect on bovine stature, identifying 8 possible candidate causal variants within *PLAG1*. Within that population of Holsteins, the candidate variants were in perfect linkage disequilibrium (LD). Using a multi-breed meta-analysis, Bouwman et al. (2018) was able to break down LD in this region of chromosome 14 and identified the single variant most highly associated with cattle stature, rs109815800 (G>T). Not only was this single-nucleotide polymorphism (SNP) the most highly associated sequence variant with cattle stature identified by Bouwman et al. (2018) but was also the most significant variant associated with cattle height reported by Utsunomiya et al. (2017) and was in perfect LD with the most significant SNP associated with growth and other pleiotropic effects, including age at puberty, reported by Fortes et al. (2013).

While the effect of the *PLAG1* mutation has been previously reported for age at puberty and height, the effect on other economically important traits such as cow stayability has not yet been explored. Here we investigate the effect of the *PLAG1* mutation on early and later in life female fertility, as well as size and growth, in a well-phenotyped Australian Brahman herd.

## Materials and Methods

Animal ethics approval was obtained from the University of Queensland ethics board, animal ethics committee approval number QAAFI/270/17.

### Animal and Herd Summary

For this study, a single herd of registered Brahman cattle in Central Queensland, Australia was used. Born between 1995 and 2018, females in this herd have been highly selected for improved fertility and early puberty, with all herd bulls ranking in the top 5% of the Australian Brahman breed for daughter fertility (BREEDPLAN, Agricultural Business Research Institute, Armidale, Australia). Heifers were first exposed to bulls at approximately 1 yr of age, with only a small proportion conceiving as yearlings (≤ 5% annually). All heifers were then exposed to bulls again at 2 yr of age. The breeding season, or joining period, begins on October 1 of each year and spans 4–5 mo, through the summer. To remain in the herd each cow must maintain a yearly calving schedule. All heifers were expected to have produced at least one calf by 3 yr of age, including those that may have first calved at 2 yr of age. Starting in 2013, pregnancy status of each female was determined via manual palpation by a trained technician approximately 5.5 mo after the start of the breeding season, and gestational maturity was recorded as fetal age in weeks.

### Phenotypes

Calf performance and cow fertility records were recorded each year as part of normal management practice, with 2,839 individual cow’s records available for this study. These records were used to generate defined heifer, lifetime fertility, and weight phenotypes, along with potential covariates, described in Table 1.

**Table 1.** Description of continuous versus binary fertility, growth, and size traits

Continuous traits	
Weeks pregnant (actual)	Fetal age in weeks recorded via manual palpation at pregnancy diagnosis
Weeks pregnant (estimated)	A combination of actual fetal age in weeks, with estimated fetal age at the average date of pregnancy diagnosis in heifers born before 2011
Heifer days to calving	Difference in days between the date of bull turn out at the beginning of the breeding season and first calving date
Age at first calving	Difference in days between first calving and date of cow’s birth
200-d weight	Heifer weight recorded at approximately weaning
400-d weight	Heifer weight recorded as approximately yearlings
600-d weight	Heifer weight recorded at approximately the start of the breeding season, at ~2 yr of age
Mature cow weight (average ≥ 3 yr)	Average of all mature cow weight measurements collected at 3 yr of age or greater
Mature cow weight (average ≥ 5 yr)	Average of all mature cow weight measurements collected at 5 yr of age or greater
Average daily gain between 200- and 400-d weights	The difference in weight measured at ~200- and 400-d of age, divided by number of days between measurements
Average daily gain between 200- and 600-d weights	The difference in weight measured at ~200- and 600-d of age, divided by number of days between measurements
Average daily gain between 400- and 600-d weights	The difference in weight measured at ~400- and 600-d of age, divided by number of days between measurements

**Binary traits** **1 = successful, 0 = unsuccessful**	
Heifer pregnancy	Ability to successfully conceive prior to 3 yr of age
Heifer calving	Ability to successfully calve by 3 yr of age
Heifer rebreed	Whether or not a heifer was able to successfully produce a calf the year following her first pregnancy
Stayability at 4 yr of age (Stay4)	Ability to produce two calves by 4 yr of age
Stayability at 5 yr of age (Stay5)	Ability to produce three calves by 5 yr of age
Stayability at 6 yr of age (Stay6)	Ability to produce four calves by 6 yr of age

A range of fertility traits were measured and recorded in the herd. Heifer pregnancy was recorded as a binary trait based upon whether each heifer was successfully able to conceive prior to 3 yr of age. Pregnancy success was determined at time of yearly pregnancy diagnosis for all heifers born in 2011 and later. For any heifers born prior to 2011, calving records were used to determine pregnancy success. Among those heifers that had both a pregnancy diagnosis and calving record available, only 6% experienced pregnancy loss after pregnancy diagnosis, making calving success a good approximation in cases where pregnancy diagnosis records were unavailable. These records were used to define heifer calving as a separate binary trait based upon a heifer’s ability to give birth to a calf by approximately 3 yr of age. Heifer rebreed was scored as a binary trait based upon whether or not a heifer was able to successfully produce a calf the year following her first pregnancy. Heifers that first calved at 2 yr of age were not considered for this trait due to limited genotyped-phenotyped records (*n* = 1).

Heifer weeks pregnant, recorded as fetal age, was used as a proxy for heifer maturity, where it was assumed that heifers that were more advanced in gestation at a common time point would have likely been more mature at the start of the breeding season than heifers that were not as advanced in their gestation. Fetal age in weeks was recorded via manual palpation at pregnancy diagnosis for all heifers born in 2011 and later. For any heifers born before this, approximate fetal age was estimated as weeks gestation at the average pregnancy diagnosis date (April 15), given recorded date of calving, and assuming a 290-d gestation length.

Days to calving is a routinely recorded trait in Australian Brahmans and is defined as the number of days between the date of bull turn out at the beginning of the breeding season and calving date. Heifer days to calving was recorded as the number of days between first calving date and date of first bull exposure. Females that did not calve as a heifer, by 3 yr of age, were not considered. Age at first calving was only available for heifers with a recorded birth date and was calculated as the difference in days between first calving date and heifer birth date. Heifers that first calved at approximately 2 yr of age were not considered due to limited genotyped-phenotyped records (*n* = 38).

Stayability is traditionally defined as a cow’s probability of surviving to a specific age given the opportunity to first reach that age (Hudson and Van Vleck, 1981). Actual herd management practices requires that a cow must have first calved at least once by 3 yr of age and then maintain a yearly calving schedule thereafter. Binary stayability was defined as a cow’s ability to produce 2 calves by 4 years of age (Stay4), her ability to produce 3 calves by 5 years of age (Stay5), and her ability to produce 4 calves by 6 yr of age (Stay6). In this herd, stayability reflects the likelihood that a cow will not be culled for poor reproductive performance prior to a given age.

Heifer weight traits were recorded at industry standard time points: 200 d of age, 400 d of age, and 600 d of age. Birth weights were not recorded, as is common in extensive northern Australia beef cattle operations. Adjusted weights were adjusted to the specified age in days following the procedures recommended by Queensland DPI (2000) and the Beef Improvement Federation (BIF, 2018), using dam age adjustments recommended by the American Brahman Breeders Association (ABBA, 2015), and assuming a standard birthweight of 30 kg. Mature cow weight was, typically, recorded on a yearly basis. On average, Australian Brahman females are expected to have reached their full mature size between 4 and 5 yr of age (Ridley and Schatz, 2006). Due to a relatively low number of records, mature cow weight was averaged following 2 time points, 3 and 5 yr of age. Heifer average daily weight gain was calculated between 200- and 400-d weights, 200- and 600-d weights, and 400- and 600-d weights. Management-based covariates were also recorded, including management cohort (either year of birth or year + month of birth groups), age at joining, and age at trait recording (pregnancy test and weighing).

### Genotyping

Genotypes of 2,839 females were generated using the Illumina Geneseek TropBeef V2 array (Neogen, Lincoln, NE). After quality control, with genotypes with QC score < 0.6 set to missing, monomorphic SNP excluded, and SNP with all heterozygous calls excluded, 50,045 SNPs were available. All genotypes were imputed to 709,000 SNPs from the Bovine HD array (following further quality control) using 4,506 cattle genotyped with the Bovine HD array (including a large number of Brahman, Droughtmaster and Santa Gertrudis cattle). Eagle (Loh et al., 2016) was used for phasing, and Minimac3 (Das et al., 2016) was used for imputation. The variant with the most significant effect on height for *PLAG1* (NC_037341.1:g.23338890G>T, rs109815800) on chromosome 14 is among those genotyped on the Bovine HD array (Utsunomiya et al., 2017; Bouwman et al., 2018). The imputed *PLAG1* genotype at this variant was isolated for each animal, with genotypes called as 0 = homozygous reference allele (GG), 1 = heterozygous (GT), and 2 = homozygous alternative allele (TT).

### Model fitting and testing

**Figure 1.**
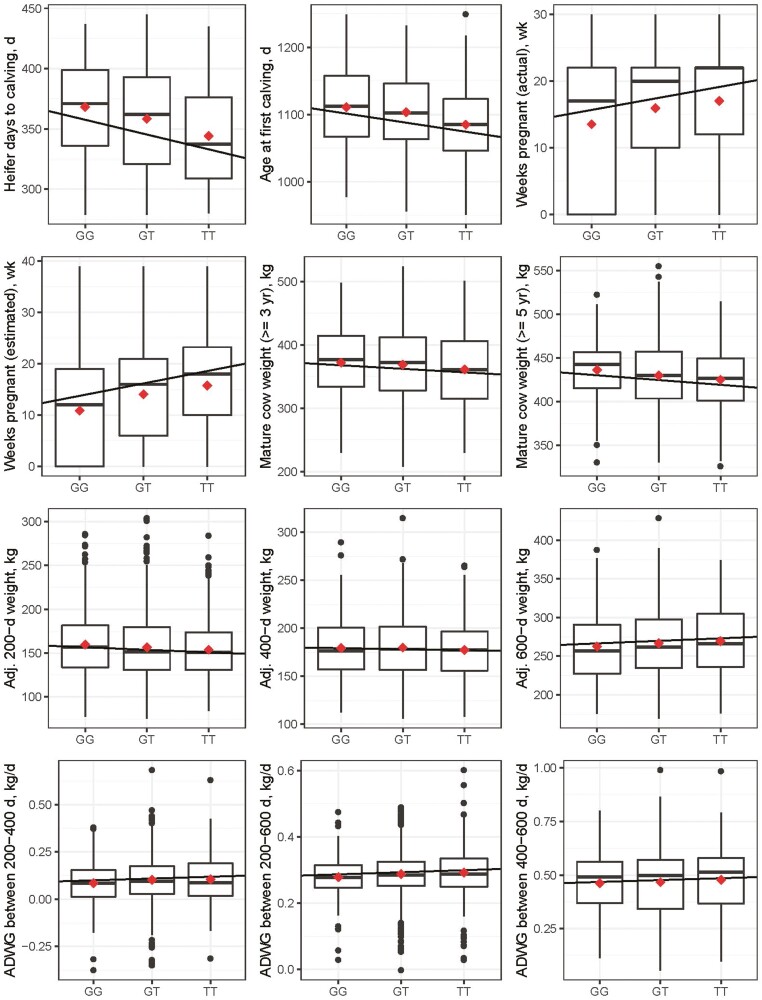
Continuous female fertility, growth, and size traits by *PLAG1* rs109815800 genotype. The trait mean is represented by the red dot for each genotype category. To improve interpretation, 200-, 400-, and 600-d weights were adjusted (adj.) to the specified age in days following the procedures recommended by Queensland DPI (2000) using dam age adjustments recommended by ABBA (2015). ADWG = average daily weight gain.

To assess the relationship between each continuous trait (Table 1, Fig. 1) and the effect of *PLAG1* genotype, a linear model was utilized using the *lm* function in R (version 3.6.2) (R Core Team, 2019). The gene action of *PLAG1* was determined by comparing models fitting *PLAG1* genotype call as either a continuous variable, assuming additive gene action (Karim et al., 2011), and as a categorical variable, to test for dominance effects (Littlejohn et al., 2012). Two phenotypes, one fertility and one weight trait, were used for this test: actual weeks pregnant (*n* = 773) and mature cow weight (averaged >3 yr of age) (*n* = 1047). The model for actual weeks pregnant included the fixed effects of *PLAG1* genotype, age at pregnancy diagnosis, and adjusted 600-d weight. The model for mature cow weight included fixed effects of *PLAG1* genotype and year of birth group. Least-squares means for each categorical genotype were calculated using the lsmeans package in R (Lenth, 2016), and a Tukey’s pairwise comparison was used to determine differences between allele combinations.

The relationship between the additive effects of *PLAG1* genotype call and the continuous traits (Table 1) were tested for significance. For each weight trait, fixed effects included the *PLAG1* genotype call (fit as a continuous variable), management cohort (year of birth group), and age at weighing (for 200-, 400-, and 600-d weights only). Adjusted weights were not used as the dependent variable.

For each continuous fertility trait (Table 1), fixed effects included *PLAG1* genotype call, fit as a continuous variable, year + month of birth group, either age at joining or age at trait measurement, and adjusted 600-d weight. Management cohort was considered as year + month of birth group, in order to account for the operation’s heifer management practices that grouped heifers by month of birth during the breeding season. Any contemporary groups with fewer than five animals were removed. Sum of squares for each fixed effect was calculated using *anova* in R (version 3.6.2) (R Core Team, 2019) and used to determine the proportion of phenotypic variance explained by *PLAG1* genotype for each trait.

To assess the relationship between each binary fertility trait (Table 1) and the additive effect of *PLAG1* genotype, a generalized linear model with a logit link was utilized, using the *glm* function in R (version 3.6.2) (R Core Team, 2019). Each binary model included the fixed effects of *PLAG1* genotype call, fit as a continuous variable, and year + month of birth group, except for Stay6 that fit the effect of management cohort as year of birth group. This was done due to the low number of Stay6 records available for each year. Only contemporary groups containing at least 3 animals of each phenotype (0 and 1) were considered. Odds ratios were calculated from the log-odds estimate for each model, and then converted to a probability scale. Proportion of model deviance explained by each fixed effect was calculated using *anova* in R (version 3.6.2) (R Core Team, 2019).

## Results

There were 2,839 animals with imputed genotypes at the putative causative loci. The *PLAG1* rs109815800 SNP was not in Hardy–Weinberg equilibrium (*P <* 0.00001). Among genotyped females, the reference allele (G) to alternate allele (T) ratio was 0.475/0.525; ~18% were homozygous for the reference allele, 60% heterozygous, and 23% were homozygous for the alternate allele. This suggests that there may be selection preference for the alternate allele within this herd.

**Figure 2.**
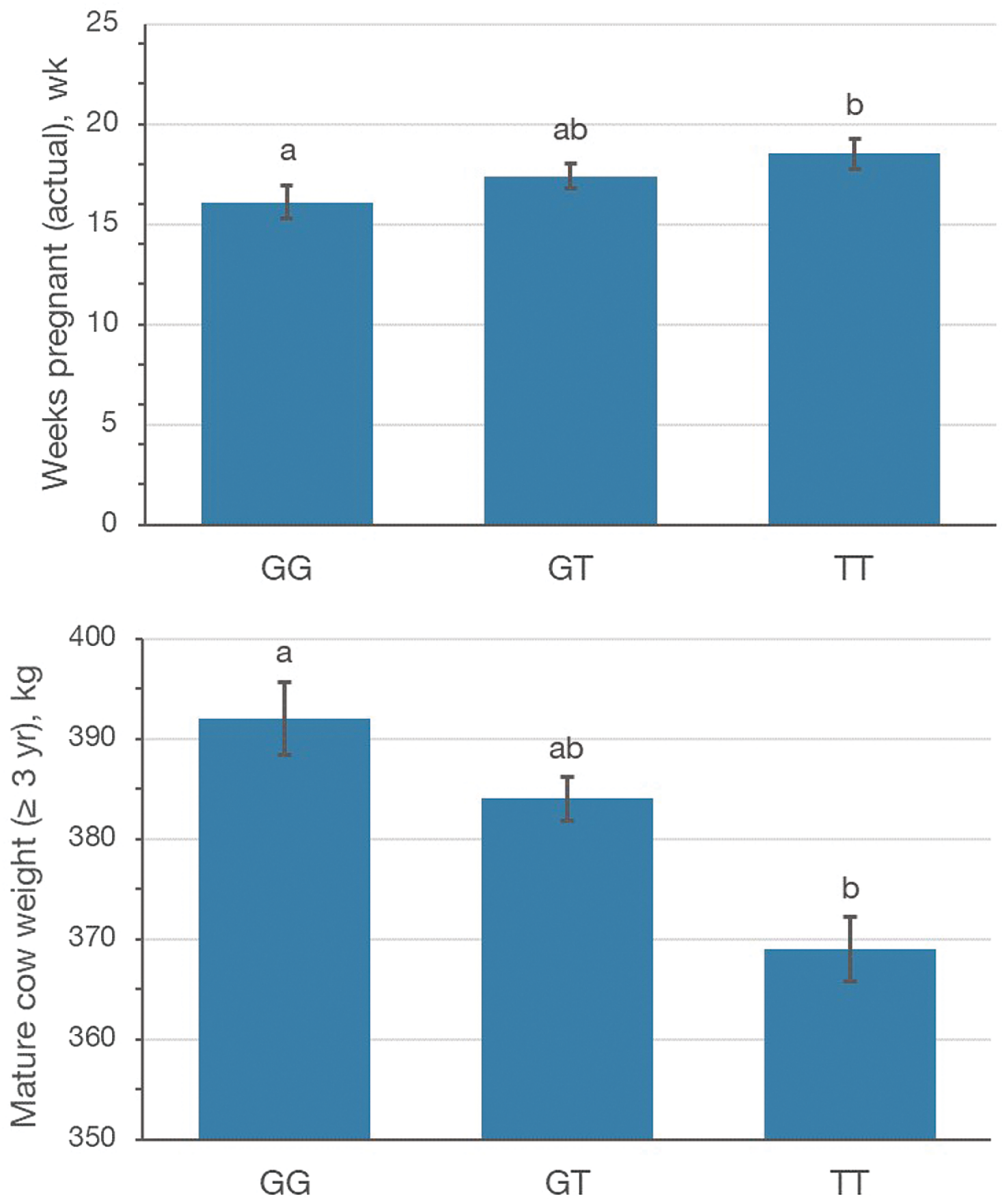
Least-squares means for continuous heifer fertility and weight traits by categorical *PLAG1* rs109815800 genotype. (A, B) Significant pairwise differences of at least *P* < 0.05.

Regardless of how heifer fertility was measured, there was a significant and desirable relationship between the additive effects of *PLAG1* genotype and successful heifer fertility (Tables 2 and 3). Heifers with 2 copies of the alternate allele (TT) conceived on average over a week sooner than contemporaries with only 1 copy (GT), and over 2 wk sooner than those with no copies of the alternate allele (GG) (Table 2). The additive effect of *PLAG1* genotype significantly increased odds of successfully conceiving and successfully calving before 3 yr of age. Interestingly, among heifers that first calved at 2 yr of age (*n* = 38), 8% were homozygous for the reference allele, 39% heterozygous, and 53% were homozygous for the alternate allele. Heifers with this genotype also have an increased odds of successfully producing 2 calves by 4 yr of age (Table 3). As the number of alternate alleles in a heifer’s genotype increases, the likelihood of successful heifer pregnancy increased by 70%. There does not appear to be a significant dominance effect of *PLAG1* genotype (Fig. 2).

**Table 2.** Additive effect of *PLAG1* genotype^1^ on continuous female fertility, growth, and size traits

**Trait**	***n***	**Additive effect of *PLAG1* genotype** ^1^	**SE**	**%Var** ^2^	**Mean**	**SD**
Weeks pregnant (actual), wk	1498	1.06*	0.44	0.61	15.74	9.44
Weeks pregnant (estimated), wk	2080	1.81**	0.39	1.51	13.83	9.80
Heifer days to calving, d	1079	−10.92***	1.78	1.76	356.89	42.12
Age at first calving, d	1110	−8.90***	1.72	0.88	1100.51	57.85
200-d weight, kg	2671	−1.76*	0.63	0.15	156.45	34.27
400-d weight, kg	1781	−1.28	0.86	0.08	179.21	30.97
600-d weight, kg	1094	0.82	1.20	0.01	266.77	44.40
Mature cow weight (≥3 yr), kg	1201	−10.88***	2.07	1.30	367.43	58.57
Mature cow weight (≥5 yr), kg	507	−9.17*	2.87	1.71	429.24	41.29
ADWG^3^ between 200 and 400 d, kg/d	1781	0.01	<0.01	0.09	0.10	0.12
ADWG^3^ between 200 and 600 d, kg/d	1071	<0.01	<0.01	0.06	0.29	0.07
ADWG^3^ between 400 and 600 d, kg/d	982	<−0.01	<0.01	<0.01	0.47	0.15

*P < 0.05; **P < 0.0001; ***P < 1 × 10^−6^.

*PLAG1* rs109815800 genotypes: 0 = homozygous reference allele (GG), 1 = heterozygous (GT), and 2 = homozygous alternative allele (TT).

%Var = percent phenotypic variance explained by *PLAG1* genotype.

ADWG = average daily weight gain.

**Table 3.** Additive effect of *PLAG1* genotype^1^ on binary^2^ female fertility traits

**Trait**	***n***	**% Success**	**Log-odds estimate**	**SE**	**%Dev** ^3^	**Odds ratio**	**95% CI**	**Prob**	**95% CI**
Heifer pregnancy	1388	0.71	0.53***	0.11	1.53	1.70	1.38–2.10	0.63	0.58–0.68
Heifer calving	933	0.66	0.47**	0.13	1.16	1.61	1.25–2.07	0.62	0.56–0.67
Heifer rebreed	596	0.66	0.11	0.14	—	—	—	—	—
*Stayability:*									
Four years	734	0.46	0.39*	0.13	0.95	1.48	1.15–1.90	0.60	0.54–0.66
Five years	379	0.38	0.32	0.19	—	—	—	—	—
Six years	259	0.59	−0.22	0.23	—	—	—	—	—

*P < 0.01; **P < 0.001; ***P < 1 × 10^−6^.

*PLAG1* rs109815800 genotypes: 0 = homozygous reference allele (GG), 1 = heterozygous (GT), and 2 = homozygous alternative allele (TT).

All binary traits; 1 = success, 0 = failure.

%Dev = percent model deviance explained by *PLAG1* genotype.

As females aged the impact of *PLAG1* genotype on fertility decreased (Table 3). Once a heifer successfully produced her first calf, there was no relationship between her *PLAG1* genotype and probability of successfully rebreeding during the subsequent breeding season. For this study, stayability was defined so that it accounted for first time heifer pregnancy success or failure. This is reflected in the significant and positive relationship between *PLAG1* genotype and Stay4, but lack of relationship between *PLAG1* and rebreed. However, as the influence of heifer pregnancy on a female’s potential to meet a stayability threshold diminished, in the cases of Stay5 and Stay6, so did the relationship between *PLAG1* genotype and trait success. This suggests that the influence of *PLAG1* is encountered early in a heifer’s life, likely on puberty, and does not directly affect her future reproductive performance.

For this study, mature cow size was assessed at 5 yr of age, and due to a relatively low number of records, also at 3 yr of age. The additive effect of *PLAG1* genotype had a negative effect on mature cow weight (*P* < 0.01), where females with 2 copies of the alternate allele (TT) were significantly smaller than those with either one or none (Table 2). A relationship between *PLAG1* genotype and growth rate was not observed, nor was there a relationship with either 400- or 600-d weight (Table 2). There was an additive effect (*P* < 0.05) of *PLAG1* genotype on 200-d weight (Table 2).

## Discussion

In this study, the effect of *PLAG1* in Australian Brahmans was greatest on puberty and early life traits, with diminishing effects on reproduction as animals aged. Females with 2 copies of the alternate *PLAG1* allele (rs109815800; T) were more moderate sized and experienced greater reproductive success as heifers. Females with 2 copies of reference *PLAG1* allele (rs109815800; G) appeared to be larger, but significantly less productive as heifers. This pattern concurs with previous reports of this mutation and haplotypes carrying this mutation in Brahman (Fortes et al., 2013), Holstein (Karim et al., 2011; Littlejohn et al., 2012), Japanese Black (Nishimura et al., 2012), and Nellore (Utsunomiya et al., 2017).

*PLAG1* was found to significantly influence size but did not have an effect on growth rate. In this Brahman herd the G allele of rs109815800 was associated with increased weaning weight, although this likely a result of the influence *PLAG1* has on fetal size and birthweight (Littlejohn et al., 2012). In absence of available birthweight records for this study, this hypothesis cannot be tested. The G allele of rs109815800 was also associated with increased mature size. This is equivalent to previous reports of increased height and weight in Holstein (Karim et al., 2011; Fink et al., 2017). *PLAG1* was previously found to not be associated with measures of growth, including feed intake, feed efficiency, residual feed intake, or Kleiber ratio in Holsteins (Fink et al., 2017), but has been associated with both greater feed intake and lower residual feed intake in other Australian Brahman animals (Fortes et al., 2013).

The allele associated with higher fertility (T) was more prevalent in this population of Australian Brahman than other Brahman populations (Fortes et al., 2013; Hayes and Daetwyler, 2019). This is consistent with this population having better fertility than other Australian Brahman herds. The higher proportion of favorable *PLAG1* genotypes in this herd may be a causal driver of their increased fertility. Additionally, the *PLAG1* allele may be increasing in this population in response to the strong selection pressure being applied, although this cannot be currently tested due to the relatively recent implementation of whole herd genotyping.

Not only is the *PLAG1* rs109815800 SNP segregating within Australian Brahman, but it also has a large association with a number of economically important production traits. While the SNP is currently included on Illumina high density cattle SNP arrays, these results suggest it should also be included on lower density SNP panels. This is especially important when genotyping indicine-influenced cattle, and should improve the accuracy of genomic breeding values for female fertility. In particular, for genomic selection schemes that fit SNP effects individually, such as Bayesian models, that can account for SNP with large effect sizes.

Evidence from prior research strongly suggests that the G allele is of taurine origin and was likely introgressed into the Brahman breed through grading-up early in breed development (Fortes et al., 2013; Utsunomiya et al., 2017; Koufariotis et al., 2018). The proportion of taurine influence in the Australian Brahman genome has been estimated between 8.94% (Koufariotis et al., 2018) and 10% (Bolormaa et al., 2011). Koufariotis et al. (2018) identified that the region in the Brahman genome with the most extreme *Bos taurus* enrichment was on chromosome 14:22-42 Mb (UMD3.1), surrounding *PLAG1*. This very closely coincides with other reports of a 20 Mb region of depressed heterozygosity containing the taurine G allele of rs109815800 that was found in Brahmans (Fortes et al., 2013). Additionally, Utsunomiya et al. (2017) reported that the haplotype containing the taurine G allele coalesced within Brahmans ~121 yBP, which is consistent with a period of formation and grading-up of the breed (American Brahman Breeders Association).

Interestingly, Koufariotis et al. (2018) observed that prominent, historical Brahman bulls (born 1953-1989) were less likely to have this taurine introgression on chromosome 14 than younger bulls (born 1990–2005). This further suggests that the taurine introgression on chromosome 14 is not only recent but is also under ongoing selection within Australian Brahmans. This is likely driven by increased selection emphasis on size. Average liveweight at maturity and slaughter in Australian beef cattle has increased by approximately 30% from 1976–2018 (Fordyce et al., 2021), and there has been a 76% increase in 600-d weight breeding values from 1999 to 2018 within Australian Brahman (ABBA, 2018). Our results suggest that an unintended consequence of this introgression may have been reduced heifer fertility in Brahmans.

Large mature cow size and female fertility often have an antagonistic relationship, potentially leading to economic losses. Vargas et al. (1999) found that Brahman heifers of large frame size were significantly less reproductively efficient than small frame heifers. Large frame heifers were significantly older at puberty and had significantly reduced calving rates, weaning rates, calf survival rates, and kilogram of calf production per cow than small frame size heifers (Vargas, 1999). Larger cows are generally more expensive, as they consume more feed on an individual basis and in many situations, the marginal increase in calf weaning weights is not adequate to overcome the higher input costs of maintaining large cows (Lalman, 2018). This is particularly important in challenging production environments, such as the tropics and sub-tropics.

In order for cattle production to be sustainable, efficient resource use must balance economic profitability and environmental impact. Reduced inputs related to improved reproductive efficiency and reduced mature cow size associated with the T allele of rs109815800 may increase the sustainability of a herd. Both measures are highly correlated indicator traits for net methane production and will impact net greenhouse gas emissions. The lower the maintenance requirement and smaller the size of the cow, the less methane she will emit (Bell et al., 2012). Cows that have more calves over their lifetime dilute their own methane emissions over more kilograms of beef. As herd fertility improves, either fewer cows can be used to produce the same amount of product or the same number of cows can produce more product without an increase in emissions (Bell et al., 2012; Quinton et al., 2018). Furthermore, the longer the cow remains in the herd, and continues to produce calves, the more she dilutes the methane she emitted as a growing heifer (Quinton et al., 2018). Selection emphasis on improved Brahman heifer fertility is likely to increase the frequency of the T allele of rs109815800, which may also increase herd profitability and long-term sustainability through improved reproductive efficiency and reduced mature cow size.

## Abbreviations:

LD: linkage disequilibrium
SNP: single-nucleotide polymorphism
